# Patient-Based Transcriptome-Wide Analysis Identify Interferon and Ubiquination Pathways as Potential Predictors of Influenza A Disease Severity

**DOI:** 10.1371/journal.pone.0111640

**Published:** 2014-11-03

**Authors:** Long Truong Hoang, Thomas Tolfvenstam, Eng Eong Ooi, Chiea Chuen Khor, Ahmand Nazri Mohamed Naim, Eliza Xin Pei Ho, Swee Hoe Ong, Heiman F. Wertheim, Annette Fox, Chau Van Vinh Nguyen, Ngoc My Nghiem, Tuan Manh Ha, Anh Thi Ngoc Tran, Paul Tambayah, Raymond Lin, Chariya Sangsajja, Weerawat Manosuthi, Chareon Chuchottaworn, Piamlarp Sansayunh, Tawee Chotpitayasunondh, Piyarat Suntarattiwong, Kulkanya Chokephaibulkit, Pilaipan Puthavathana, Menno D. de Jong, Jeremy Farrar, H. Rogier van Doorn, Martin Lloyd Hibberd

**Affiliations:** 1 Genome Institute of Singapore, Singapore, Singapore; 2 The Hospital for Tropical Diseases, Wellcome Trust Major Overseas Programme, Oxford University Clinical Research Unit, Ho Chi Minh City, Vietnam, and Centre for Tropical Medicine, Nuffield Department of Medicine, University of Oxford, Oxford, United Kingdom; 3 Department of Medical Microbiology, Academic Medical Center, University of Amsterdam, Amsterdam, The Netherlands; 4 National Hospital of Tropical Diseases, Wellcome Trust Major Overseas Programme, Oxford University Clinical Research Unit, Hanoi, Vietnam, and Centre for Tropical Medicine, Nuffield Department of Medicine, University of Oxford, Oxford, United Kingdom; 5 Hospital for Tropical Diseases, Ho Chi Minh City, Vietnam; 6 Children Hospital 2, Ho Chi Minh City, Vietnam; 7 National University Hospital, Singapore, Singapore; 8 Bamrasnaradura Infectious Disease Institute, Nonthaburi, Thailand; 9 Chest Disease Institute, Nonthaburi, Thailand; 10 Queen Sirikit National Institute of Child Health, Bangkok, Thailand; 11 Faculty of Medicine Siriraj Hospital, Mahidol University, Bangkok, Thailand; 12 Infection Immunology, Respiratory Infections, Karolinska Institutet, Solna, Sweden; Erasmus Medical Center, Netherlands

## Abstract

**Background:**

The influenza A virus is an RNA virus that is responsible for seasonal epidemics worldwide with up to five million cases of severe illness and 500,000 deaths annually according to the World Health Organization estimates. The factors associated with severe diseases are not well defined, but more severe disease is more often seen among persons aged >65 years, infants, pregnant women, and individuals of any age with underlying health conditions.

**Methodology/Principal Findings:**

Using gene expression microarrays, the transcriptomic profiles of influenza-infected patients with severe (N = 11), moderate (N = 40) and mild (N = 83) symptoms were compared with the febrile patients of unknown etiology (N = 73). We found that influenza-infected patients, regardless of their clinical outcomes, had a stronger induction of antiviral and cytokine responses and a stronger attenuation of NK and T cell responses in comparison with those with unknown etiology. More importantly, we found that both interferon and ubiquitination signaling were strongly attenuated in patients with the most severe outcomes in comparison with those with moderate and mild outcomes, suggesting the protective roles of these pathways in disease pathogenesis.

**Conclusion/Significances:**

The attenuation of interferon and ubiquitination pathways may associate with the clinical outcomes of influenza patients.

## Introduction

The influenza A virus is an enveloped-, single-stranded, segmented negative-sense RNA virus that is responsible for seasonal epidemics worldwide. The World Health Organization estimates that seasonal influenza results in up to five million cases of severe illness and 500,000 deaths annually. The risk factors associated with severe diseases are not well defined, but more severe disease is more often seen among persons aged >65 years, infants, pregnant women, and individuals of any age with underlying health conditions [1].

The interaction between the virus and the host genetic and immune status clearly plays an important role in determining the outcome of the infection [2]. In human and animal models of seasonal influenza, influenza virus A/H5N1 (Avian Influenza A H5N1 virus) and A/H1N1/1918 (Influenza A H1N1 1918 strain) the cytokine levels in host respiratory secretions and serum have been associated with disease severity and outcome [3]. Hypercytokinemia was observed in humans infected with influenza virus A/H5N1 and found to be correlated to severity and mortality [4]. The innate immune responses in humans infected with influenza virus A/H5N1 suggest that pro-inflammatory mediators are contributing to disease pathogenesis, with elevated blood levels of IL-6, TNF-α, IFN-γ, and sIL-2R and elevated levels of IP-10, MCP-1, and MIG observed in patients [5]. Such responses may contribute to acute lung injury, acute respiratory distress syndrome (ARDS) and multi-organ failure observed in many patients. Recently, IFITM3, an interferon-induced transmembrane protein, has been shown to have important roles in restricting the morbidity and mortality in mice infected with influenza virus [6].

The majority of host response studies in influenza have focused on direct measurements of immunological markers in blood from patients or from expression profiles in lung tissue in animal models [3], [7], [8]. However, studies that have assessed the functional interactions between gene expression profiles and individual clinical presentation have not been performed. In this prospective study we explored these functional interactions and linked these data with the clinical features of patients infected with influenza viruses in various degrees of severity to provide insights into the pathogenesis of severe influenza.

## Materials and Methods

### Patient population and sample collection

**Mild influenza samples and other febrile illness (OFI) samples.** Patients were recruited between January 2008 and January 2010 from an undifferentiated fever inclusion study (EDEN) in Singapore, focusing on early enrollment after fever development. The EDEN study has been ongoing since 2005 [15]. Individuals eligible for inclusion gave their written consent to participate in the study, were ≥18 years of age and presented ≤72 h from onset of fever ≥38°C. Patients were tested by PCR for influenza A and B, respiratory syncytial virus, parainfluenza 1–3, coronavirus, metapneumovirus, enterovirus and adenovirus in nasal swabs and for dengue virus 1–4, human parvovirus B19, Cytomegalovirus, and Epstein Bar virus in EDTA blood. To characterize the early transcriptional response to influenza A infection *in-vivo*, we performed whole-blood transcriptional profiling on all samples from both groups at 72 h after fever onset, at 3–8 days and 3–4 weeks after self-reported fever onset. The time point of the 1st, 2nd and 3rd sampling was regarded as acute disease, defervescence and convalescence, respectively.

#### Moderate and severe influenza samples

Samples of moderate and severe influenza patients were collected from a multi-center, double-blinded, randomized control trial of standard dose (75 mg bd or pediatric equivalent) versus double dose (150 mg bd or pediatric equivalent) oseltamivir for the treatment of influenza patients requiring hospitalization (Registered ClinicalTrials.gov: NCT00298233) [9]. The study took place across five hospitals in Vietnam, three hospitals in Indonesia, four hospitals in Thailand, and one in Singapore, all hospitals being part of the Southeast Asia Infectious Disease Clinical Research Network (SEAICRN). The primary endpoint of the trial was the proportion of subjects with no detectable viral RNA in respiratory swabs at day 5 as measured by RT-PCR. Whole-blood samples were collected in PAXgene tubes on day 0, 5 and 28 of enrolment into the study. For this study, to avoid the effect of the treatment on the host response, only the whole blood samples collected before oseltamivir treatment (study day 0) and at follow up (day 28) were used.

The inclusion criteria of this study were: (i) age ≥1 year, (ii) duration of illness ≤10 (non-H5N1) or ≤14 (H5N1) days, (iii) positive result for influenza virus A or B using a rapid antigen test or qualitative reverse transcriptase polymerase chain reaction (RT-PCR) in a respiratory specimen, (iv) presence of at least one respiratory symptom (cough, dyspnea or sore throat), (v) disease requiring hospital admission, and (vi) one of the following signs of severe influenza: (a) new infiltrate on a chest X-ray, (b) tachypnea (respiratory rate ≥30 for ages ≥12 years, rate ≥40 for ages 6 to 12 years, rate ≥45 for ages 3 to 6 years, rate ≥50 for ages 1– to 3 years), (c) dyspnea (unable to speak full sentences, or use of accessory respiratory muscles), or (d) hypoxia (arterial oxygen saturation ≤92% on room air by a transcutaneous method). Subjects infected with avian influenza virus A/H5N1 were enrolled with any degree of severity. The exclusion criteria were: (i) pregnancy or urine β-hCG positivity, (ii) breast feeding, (iii) prior oseltamivir therapy for >72 hours duration or double dose (any duration) within the past 14 days, (iv) allergy or severe intolerance of oseltamivir, (v) creatinine clearance (CrCl) <10 mL/min. Severe influenza was defined as: i) requiring mechanical ventilation or ii) presenting with severe tachypnea ((respiratory rate ≥30 for ages ≥12 years, rate ≥40 for ages 6 to 12 years, rate ≥45 for ages 3 to 6 years, rate ≥50 for ages 1–to 3 years)) and hypoxia (arterial oxygen saturation ≤92% on room air by a transcutaneous method.

### Ethics statement

The EDEN study was conducted in accordance with the Declaration of Helsinki and approved by the National Health Group (NHG) ethical review board (DSRB B/05/013). Individuals eligible for inclusion gave their written consent to participate in the study, and were ≥18 years of age. Samples of moderate and severe influenza patients were collected from a multi-center, double-blinded, randomized control trial of standard dose (75 mg bd or pediatric equivalent) versus double dose (150 mg bd or pediatric equivalent) oseltamivir for the treatment of influenza patients requiring hospitalization (Registered ClinicalTrials.gov: NCT00298233) [9].

### Gene expression microarray

One-color array technology on the Illumina platform (Illumina Inc, San Diego, CA, USA) was used for gene expression microarray. In brief, whole-blood (2.5 ml) from PAXgene RNA tubes (Qiagen, Sussex, UK) was extracted using Paxgene RNA kits (Qiagen). Biotinylated amplified cRNA was generated by in vitro transcription (IVT) technology using Illumina TotalPrep RNA Amplification Kit (Ambion, Inc., Austin, TX, USA) according to the manufacturer’s instructions. After purification, 2 µg of cRNA was hybridized to an Illumina HumanRef-12 V4 BeadChip (containing probes to more than 29,000 gene transcripts) at 55°C for 18 hours following the manufacturer’s instructions (Illumina, Inc., San Diego, CA, USA). This was followed by washing, blocking and streptavidin-Cy3 staining steps. Finally, the chip was scanned with an Illumina Bead Array Reader confocal scanner and checked using Illumina QC analysis. Background subtracted raw gene expression intensity data was exported from the GenomeStudio software and used for further analysis.

### Data normalization

All datasets were normalized by using R (http://www.bioconductor.org) as described below. First, the raw data was log10 transformed before Z score transformation was performed [10]. The Z score was calculated for each sample by subtracting the overall mean gene intensity from the raw intensity signal for each gene (reference). After that, this data was divided by the standard deviation of all of the measured intensities as in the following formula:
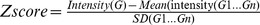
where G is any gene on the microarray and G1…Gn represents the aggregate measure of all of the genes.

### Statistical analysis

We used the Z score as the base value to identify differentially expressed probes in comparisons between any two groups of samples. Probes with high Z score are those highly expressed while those with low Z score are the least expressed probes [10]. Conventional fold change calculation could be simple to understand but at low intensities, where data is much more variable, false positive rate could increase. In contrast, at high intensity, where probes are significantly expressed might not be identified. Z score transformation calculate the number of standard deviations a particular data point is form the mean, To identify differentially expressed probes in each group of patients, Z ratio for each genes was calculated [10]. Z score was calculated by dividing the mean difference in Z score between the groups by the standard deviation of the Z score difference across all the genes.
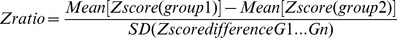
Where G1…Gn represents the aggregate measure of all the genes. A Z ratio of ± 1.96 is equivalent to the significant level of P value<0.05 [10].

### SAM

SAMR package for R (www.bioconductor.org) was used for the Z-score normalized data. For comparison between acute and convalescent samples from OFI, mild and moderate patients, two class-paired test was used. For comparison between acute and convalescent samples from patients with severe influenza, a two class-unpaired test was used. The SAM procedure combines the calculation of a t-test statistic value for each gene with subsequence permutation (N = 1,000) analysis and the calculation of false discovery rate (FDR). Statistic data from Z difference, Z ratio and SAM were combined to identify the differentially expressed genes. Significant genes were those that have SAM FDR ≤0.05, Z ratio of ± 1.96.

### Ingenuity Pathway analysis (IPA)

The differentially expressed genes (DEGs) were identified using Ingenuity Pathway Analysis (IPA) (www.ingenuity.com). The IPA database contains canonical pathways and functional gene relationships expertly-curated from the literature which helps us to understand the disease processes by identifying key biological functions and novel molecular networks. DEG lists are cross-referenced against this database to identify enriched pathways associated with the study conditions. Significant canonical pathways were defined as having a Fisher’s exact test P value<0.05 (B–H correction). In addition to filtering by the P values, each enriched pathways were analyzed carefully by taking into account the ratio and the number of the gene in each pathway.

## Results

### Mild and OFI (Other Febrile Illness) patients

A total of 167 patients with flu-like symptoms were recruited into the study. Of these 167 patients, 84 were infected with influenza virus A/H1N1pdm09 (Influenza A H1N1 pandemic 2009), pre-2009 seasonal A/H1N1, H3N2 virus, rhinovirus, or co-infected with rhinovirus and seasonal H1N1 virus. The other 83 were all pathogen-negative patients during the same time interval and were therefore designated as the OFI group. Amongst the 84 pathogen-infected patients, those who were infected with rhinovirus or co-infected with rhinovirus were removed. Only patients infected with influenza A virus were used for the data analysis (N = 73). These patients were defined as having mild influenza because they were not hospitalized and did not present with any symptoms of moderate or severe influenza. To match the duration of illness of moderate and severe patients, mild influenza samples collected at the second time point (ranging from day 3 to day 8) and their follow-up samples (3–4 weeks after) were used. The baseline characteristics of these patients are summarized in **Table 1** and their clinical symptoms are summarized in **Table 2**.

**Table 1:** Baseline characteristics of the patients in the study.

Symptoms	OFI (N = 83)	Mild (N = 73)	Moderate (N = 40)	Severe (N = 11)
**Demographic**				
Age, years median (range)	25 (18–70)	25 (18–69)	41.5 (5–70)	24 (19–73)
Sex, Female (%)	26 (31.3%)	24 (32.9%)	21 (52.5%)	4 (36%)
D.O.I, day median (range)	6 (3–8)	5 (3–8)	4 (1–9)	6 (2–9)
**Virology**				
Quantitative PCR positive		ND	**35 (86%)**	**6 (55%)**
Influenza PCR Ct value		ND	31.52 (21.1–39.9)	34.825 (26.1–39.88)
2009 pandemic H1N1	Neg	9 (12%)	24 (60%)	6 (54.4%)
H1N1	Neg	45 (62%)	1 (2.5%)	2 (18.2%)
H3N2	Neg	19 (26%)	14 (35%)	3 (27.4%)
H5N1	Neg	0 (0%)	1 (2.5%)	0 (0%)
**Hematology, median (range)**				
WBC (10^3^/µL)	6.1 (1.3–18.9)	4.7 (1.4–9.5)	5.9 (1.55–6.44)	**11.89 (4.6–28)**
Neutrophil (10^3^/µL)	3.45 (1.3–9.4)	2.6 (0.5–6.5)	3.67 (0.62–10.88)	**9.6 (2.1–24.5)**
Lymphocyte (10^3^/µL)	2 (0.7–6.1)	1.7 (0.5–3.6)	2.19 (0.23–7.56)	3 (0.24–11.57)
HGB (g/dL)	14.6 (6.9–23.7)	14.85 (9.5–22.4)	13 (10.7–16.2)	11.7 (8.7–15.2)
HCT (%)	43.5 (21.9–81.1)	44.6 (29.8–71.6)	38.8 (32.5–44.9)	35.1 (27.8–46)
PLT (10^3^/µL)	273 (86–513)	218 (79–370)	206 (55–345)	115 (27–250)

Values are presented in median (range); WBC: white blood cell count (normal range: 4–12×10^3^/mm^3^); OFI: other febrile illness; D.O.I: day of illness; HGB: hemoglobin; HCT: hematocrit; PLT: platelet; ND: not done; Neg: negative.

**Table 2:** Clinical manifestations of the patients in the study.

Symptoms assessed in all patients	OFI(N = 84)	Mild(N = 73)	Moderate(N = 40)	Severe(N = 11)
Hospitalization, case (percentage)	0 (0%)	0 (0%)	40 (100%)	11 (100%)
Headache, case (percentage)	24 (29%)	20 (27%)	26 (65%)	7 (64%)
Diarrhea, case (percentage)	2 (2.4%)	3 (4%)	11 (28%)	2 (18%)
Nausea, case (percentage)	8 (10%)	3 (4%)	13 (33%)	2 (18%)
Vomiting, case (percentage)	1 (1.2)	0 (%)	15 (38%)	2 (18%)
cough, case (percentage)	23 (28%)	28 (38%)	39 (98%)	11 (100%)
Sore throat, case (percentage)	9 (11%)	6 (8%)	24 (60%)	5 (45%)
**Symptoms that were assessed only in moderate and severe patients**				
Admitted to Intensive care unit, count (percentage)	–	–	**7 (17.5%)**	**11 (100%)**
Supplemental Oxygen, count (percentage)	–	–	**17 (43%)**	**11 (100%)**
Arterial Oxygen Saturation <92%, count (percentage)	–	–	4 (10%)	2 (18%)
Mechanical Ventilation, count (percentage)	–	–	**0 (0%)**	**7 (64%)**
Severe Dyspnea, count (percentage)	–	–	**1 (2.5%)**	**7 (63%)**
Respiratory Rate, median (range)	–	–	22 (14–40)	30 (20–38)
Severe Tachypnea, count (percentage)	–	–	**2 (5%)**	**6 (55%)**
Temp, median (range)	–	–	38 (36–40.4)	39 (37.9–40.2)
Pulse, median (range)	–	–	90 (68–120)	118 (70–155)
Blood Pressure, median (range)	–	–	111.5 (90–160)	105 (60–140)
Lansky Score, median (range)	–	–	80 (50–100)	20 (10–60)
Abnormal Cardiovascular, count (percentage)	–	–	**1 (2.5%)**	**5 (45%)**
Coma, count (percentage)	–	–	0 (0%)	1 (9%)
Crackles, count (percentage)	–	–	**22 (55%)**	**9 (82%)**
Abnormal X-ray, count (percentage)	–	–	**31 (78%)**	**11 (100%)**
Pleural Effusion, count (percentage)	–	–	0 (%)	2 (18%)

### Moderate and severe influenza patients

Samples from the moderate and severe influenza patients were collected during the same time frame (from 2007 to 2010), and a total of 153 whole blood samples from 51 patients were collected. Of these 51 patients, 30 patients were infected with influenza virus *A/H1N1pdm09*, 17 patients with *A/H3N2* virus, and three with pre-2009 *seasonal A/H1N1* and one with *A/H5N1* virus. Among the 51 patients, only 11 fulfilled the criteria for severe disease while the 40 remaining patients were considered to have moderate disease. Although the sample sizes are small, there does not appear to have any correlation between severity and the infecting influenza virus strain. The baseline characteristics of both groups of patients are summarized in **Table 1**.

At the first time point, patients with severe disease were enrolled after a median duration of illness of 5.8 days (interquartile range or IQR of 2–9 days), and patients with moderate disease after 4.3 days (IQR 1–9 days); the distribution in days of illness prior to enrolment was not statistically significant between both groups. At enrolment, severe patients had significantly higher total white blood cell counts (**Table 1**). When the differential white cell counts were scrutinized, patients with severe disease had significantly higher absolute neutrophil counts. The absolute platelet count was also significantly lower in the patients with severe disease.

### Gene expression microarray

**Figure 1:**
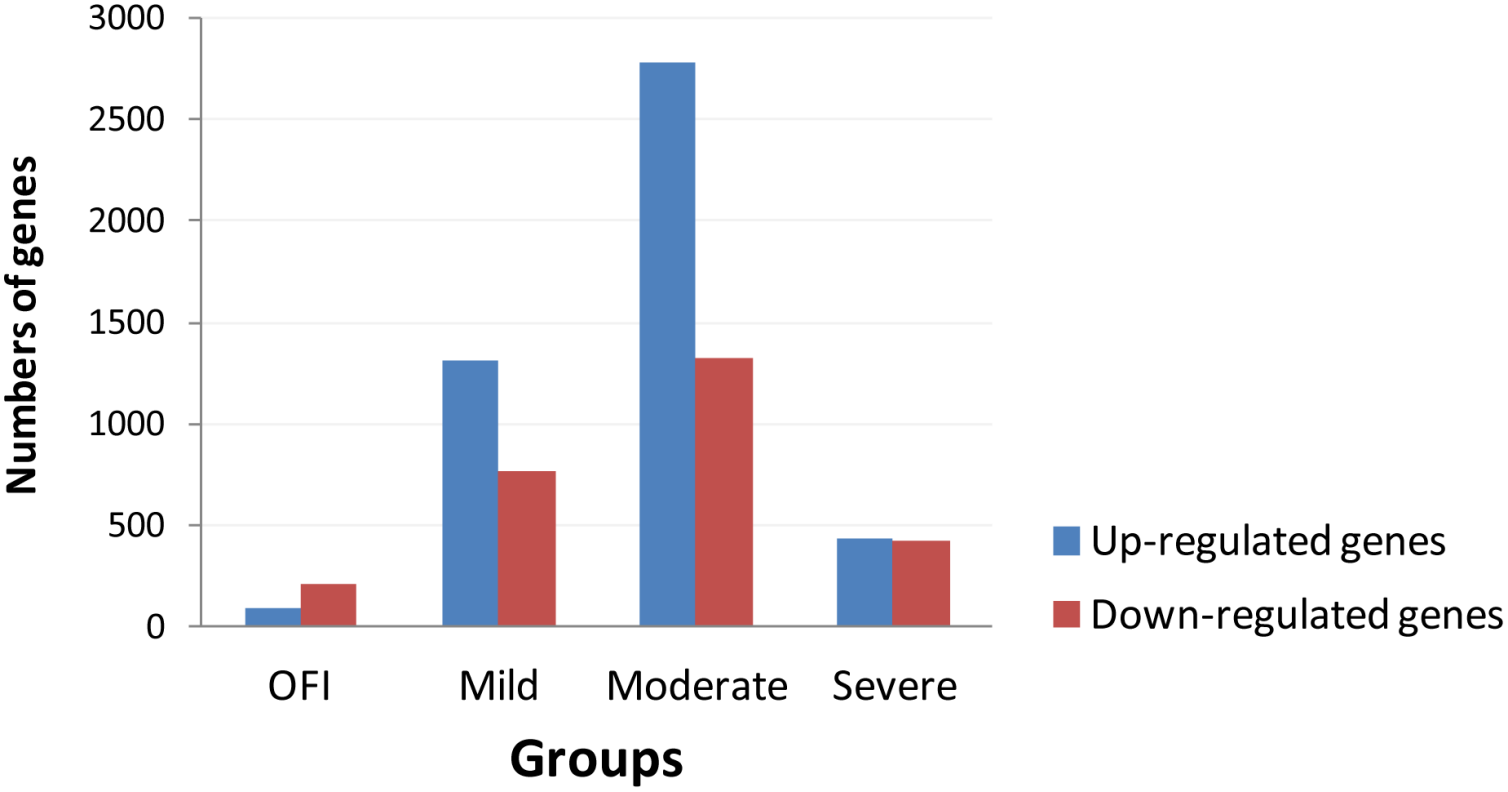
The numbers of differentially expressed transcripts (FDR 0.05, fold change >2) were observed in patients with mild and moderate influenza in comparison with OFI and severe patients. The y-axis shows the number of differentially expressed transcripts in acute samples for each condition on the x-axis in comparison with their convalescent samples. Up-regulated genes in the acute phase are in blue, genes down-regulated in dark red.

Using paired SAM test to compare expression profiles of acute (N = 83) and convalescent (N = 83) samples from OFI patients, we identified 287 differentially expressed transcripts (DATs). Of these 287 transcripts, 201 were less abundant and 86 were more abundant in acute samples. Using the same criteria, we detected 2,081 (1,316 more and 765 less abundant) DATs in acute samples from patients with mild influenza in comparison with their convalescent samples. When comparing acute samples from moderate patients with the convalescent (N = 40), we identified 4,108 DATs (2788 more and 1,320 less abundant). For severe patients, because follow-up samples were available for only 7 out of 11 patients, we performed unpaired SAM test instead of paired SAM test in order to avoid losing samples. For this analysis, we found 854 DATs in acute samples (430 more and 424 were less abundant). The results of these comparisons are summarized in **Figure 1**.

#### Up-regulated pathways

**Figure 2:**
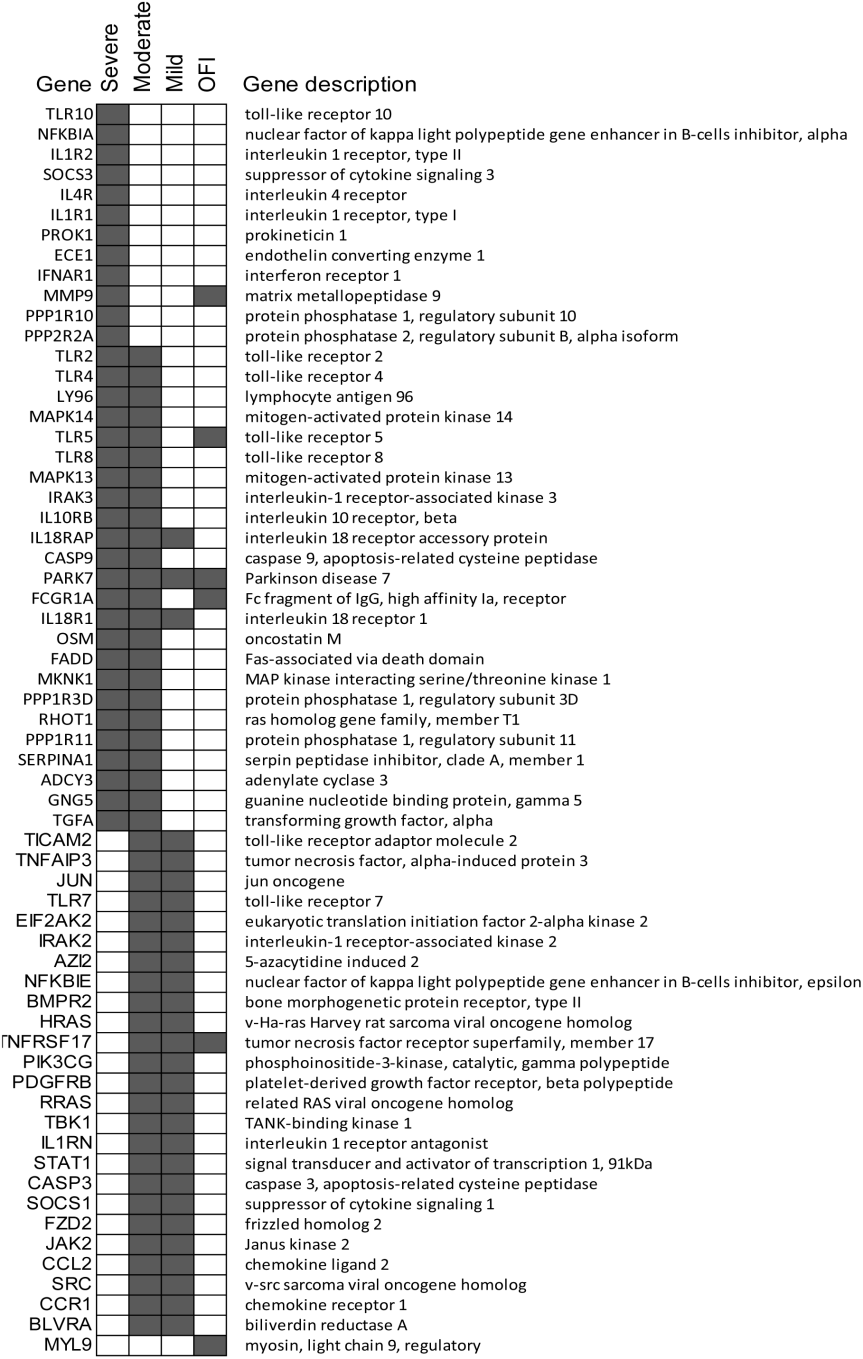
Genes that were involved in Toll-like receptor signaling, IL-10 signaling, Role of PKR in Interferon Induction and Antiviral Response and NFkB signaling pathways. The pathway names were shared between different groups but the activated genes in each pathway were different. Differentially expressed genes (FDR <0.05, fold change >2) were highlighted in grey.

**Figure 3:**
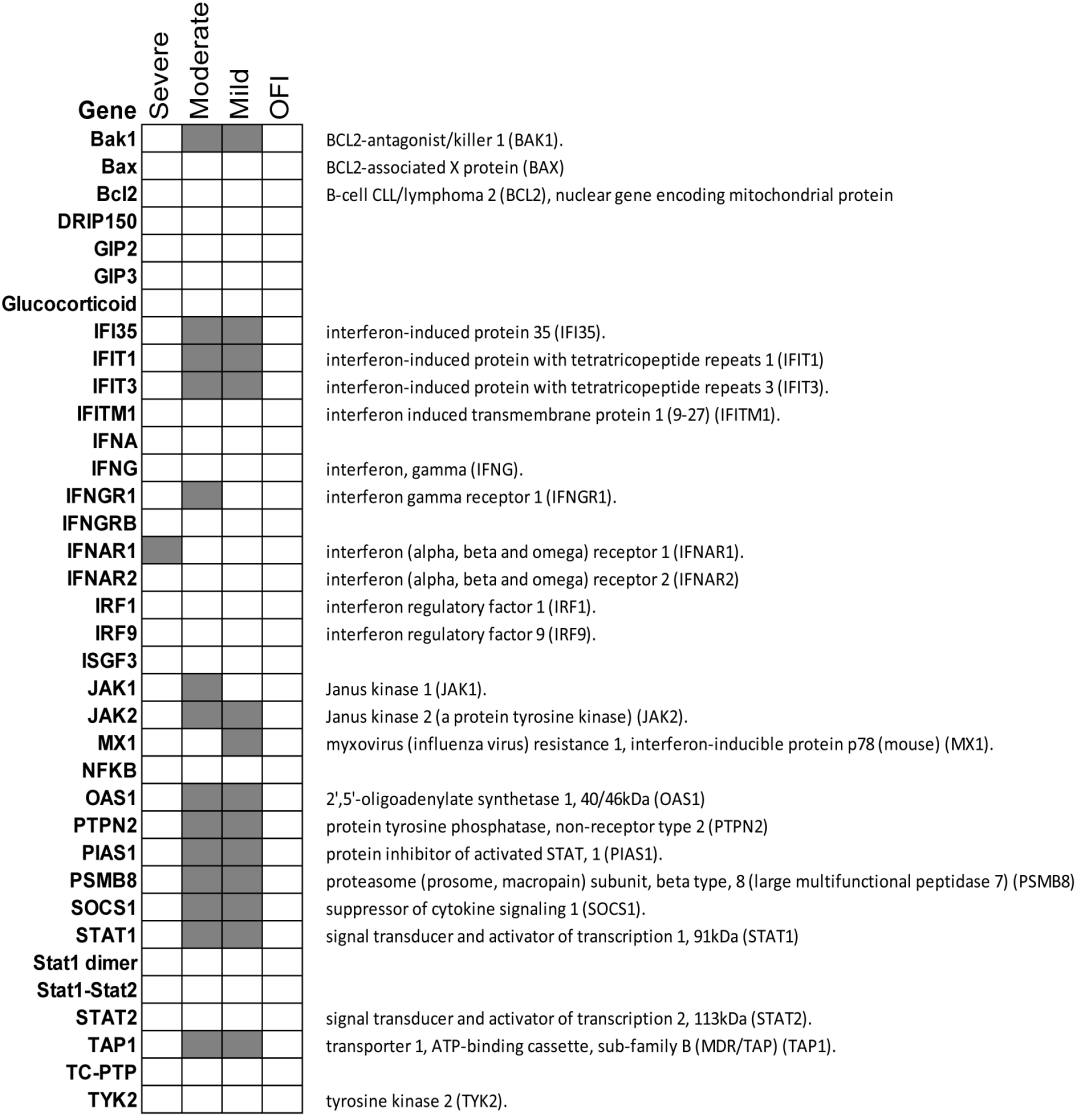
Interferon signaling pathways was highly up-regulated in moderate and mild influenza patients but was attenuated in patients with severe outcome. Up-regulated genes were highlighted in grey. IFNGR1 was the only gene that was up-regulated in severe patients while a large number of other genes were up-regulated in moderate and mild patients.

In comparison with febrile patients with unknown aetiology, patients with influenza infections showed strong antiviral and cytokine responses. Amongst the most significant pathways, Toll-like receptor signaling, IL-10 signaling, Role of PKR in Interferon Induction and Antiviral Response and NFkB signaling were significantly up-regulated in the influenza patients. Significant genes associated with these pathways in each condition are summarized in **Figure** **2**. Although the same pathways were activated in influenza patients regardless of their severity, different genes sets in each pathway were activated in patients with different outcomes (**Figure 2**). For example, patients with severe influenza had significant up-regulation of TLR10, NFKBIA, IL1R2, SOCS3, IL4R, IL1R1, PROK1, ECE1, IFNAR1, MMP9, PPP1R10 and PPP2R2A whilepatients with moderate influenza shared similar antiviral and cytokine response with both those with severe (TLR2, TRL4, TLR5, TLR8, IL10RB, IL18RAP, IL18R1, MAPK13, MAPK14, FCGR1A and IRAK3) and with mild outcomes (TLR7, TICAM3, IL1RN, STAT1, SOCS1, JAK2, IRAK2, TNFAIP, CASP3, CCL2 and CCR1). Genes in IL-1 signaling, IL-22 signaling, Production of Nitric Oxide and Reactive Oxygen Species in Macrophages and p38 MAPK signaling were only up-regulated in moderate and severe patients (**Table 3**). In comparison with patients with severe outcome, those with moderate and mild outcome were characterized with a significant up-regulation of protein ubiquitination, interferon signaling pathway and Activation of IRF by Cytosolic Pattern Recognition Receptors (**Table 4**). The interferon signaling pathway was completely attenuated in patients with severe influenza (only one gene was activated: IFNGR1) whereas the pathway was strongly up-regulated in patients with moderate (P = 10^−2.8^, ratio 0.36, 13 genes) and mild (P = 10^−4.8^, ratio = 0.33, 12 genes) outcomes (**Figure 3**). Similarly, the protein ubiquitination pathway were highly up-regulated in moderate (P = 10^−9^, ratio 0.26, 63 genes) and mild (P = 10^−7^, ratio 0.16, 42 genes) patients but was not up-regulated in those with severe outcome (**Table 4**).

**Table 3:** Canonical pathways that were up-regulated in patients with severe, moderate and mild influenza.

Ingenuity Canonical Pathways	Severe			Moderate			Mild			OFI		
	P	Ratio	# genes	P	Ratio	# genes	P	Ratio	# genes	P	Ratio	# genes
Toll-like Receptor Signaling	4.7	0.16	10	5.8	0.36	23	0.4	0.09	6	0.8	0.02	1
IL-10 Signaling	3.2	0.12	9	2.1	0.23	18	0.3	0.08	6	NS	0.00	0
Hepatic Fibrosis/Hepatic Stellate Cell Activation	2.2	0.07	10	NS	0.00	0	0.1	0.05	8	1.1	0.01	2
iNOS Signaling	2.0	0.11	6	2.8	0.28	15	0.4	0.09	5	NS	0.00	0
Parkinson’s Signaling	2.0	0.21	4	1.0	0.26	5	0.3	0.11	2	1.3	0.05	1
Role of Macrophages,Fibroblasts and EndothelialCells in Rheumatoid Arthritis	2.0	0.05	10	2.0	0.16	53	0.1	0.05	17	0.6	0.01	2
Phosphatidylglycerol Biosynthesis II	2.0	0.12	4	0.6	0.12	4	NS	0.00	0	NS	0.00	0
p38 MAPK Signaling	1.9	0.08	9	1.3	0.18	22	NS	0.00	0	NS	0.00	0
Production of Nitric Oxide and Reactive Oxygen Species in Macrophages	1.7	0.05	10	1.4	0.15	31	NS	0.00	0	NS	0.00	0
Role of PKR in Interferon Induction and Antiviral Response	1.6	0.10	5	1.9	0.25	12	0.3	0.08	4	0.9	0.02	1
IL-22 Signaling	1.6	0.16	4	0.9	0.24	6	NS	0.00	0	NS	0.00	0
NF-κB Signaling	1.6	0.06	10	3.0	0.21	38	0.4	0.08	14	0.9	0.01	2
Role of JAK family kinases in IL-6-type Cytokine Signaling	1.6	0.14	4	2.3	0.36	10	0.3	0.11	3	NS	0.00	0
IL-1 Signaling	1.4	0.06	7	1.3	0.17	18	NS	0.00	0	NS	0.00	0

P: P value was identified using Fisher’s Exact test and corrected by Benjamini Hochberg multiple testing correction; Ratio: The proportion of differentially expressed genes in a pathway; #gene: Number of differentially expressed genes in a pathway; NS: not significant; OFI: Other febrile illness.

**Table 4:** Canonical pathways that were up-regulated only in patients with moderate and mild outcomes.

Ingenuity Canonical Pathways	Severe			Moderate			Mild			OFI		
	P	Ratio	# genes	P	Ratio	# genes	P	Ratio	# genes	P	Ratio	# genes
Protein ubiquitination pathway	0.2	0.02	6	9.5	0.26	69	7.3	0.16	42	1.3	0.01	3
Cell Cycle Control of Chromosomal Replication	NS	0.00	0	3.0	0.35	12	5.7	0.35	12	4.0	0.09	3
Interferon Signaling	0.2	0.03	1	2.8	0.36	13	4.8	0.33	12	NS	0.00	0
Mitotic Roles of Polo-Like Kinase	NS	0.00	0	2.0	0.23	17	2.3	0.18	13	4.1	0.05	4
Salvage Pathways of Pyrimidine Deoxyribonucleotides	NS	0.00	0	1.8	0.24	5	2.3	0.24	5	1.5	0.05	1
Activation of IRF by Cytosolic Pattern Recognition Receptors	0.4	0.04	3	1.9	0.22	16	2.0	0.16	12	NS	0.00	0
Hereditary Breast Cancer Signaling	0.2	0.02	3	2.0	0.19	25	1.5	0.12	16	NS	0.00	0
p53 Signaling	0.3	0.03	3	4.3	0.26	29	1.5	0.12	14	NS	0.00	0
Role of CHK Proteins in Cell Cycle Checkpoint Control	0.3	0.03	2	1.5	0.22	13	1.5	0.17	10	NS	0.00	0
Regulation of Cellular Mechanics by Calpain Protease	NS	0.00	0	1.4	0.18	13	1.5	0.14	10	NS	0.00	0
ATM Signaling	1.1	0.08	5	3.0	0.29	19	1.4	0.15	10	0.7	0.02	1
Role of BRCA1 in DNA Damage Response	0.3	0.03	2	1.5	0.20	14	1.4	0.14	10	NS	0.00	0

P: P value was identified using Fisher’s Exact test and corrected by Benjamini Hochberg multiple testing correction; Ratio: The proportion of differentially expressed genes in a pathway; #gene: Number of differentially expressed genes in a pathway; NS: not significant; OFI: Other febrile illness.

Many interferon-induced transmembrane (IFITM) proteins were shown to play important roles in influenza disease outcomes. Here, we investigated all the IFITMs and other interferon-induced genes (64 in total) that were included on the Illumina HumanRef-12 V4 BeadChip. Table 5 shows all the IFITMs and other interferon-related genes and their expression levels in our patient groups. Amongst the 72 transcripts, 30 transcripts were significantly up-regulated in patients with moderate and mild outcomes while none of these were significant in patients with severe outcome or those with OFI (Table 5). Amongst the most significant genes are IFI27, IFI44, OAS3, OAS1, OASL, IFIT1, IFIH1, IFIT3 and DHX58 (RIG-I) which were highly up-regulated in moderate and mild patients and down-regulated in patients with severe outcome (**Table 5**).

**Table 5:** Expression difference of interferon-induced transmembrane (IFITM) proteins and other interferon-induced genes in the 4 different groups of patients.

ILMN Gene	Entrez ID	Moderate		Mild		Severe		OFI	
		Log Ratio	FDR	Log Ratio	FDR	Log Ratio	FDR	Log Ratio	FDR
IFI27	3429	2.53	<0.05	3.22	<0.05	0.47	49.13	0.79	<0.05
IFI44	10561	1.48	<0.05	1.27	<0.05	−0.08	NaN	0.21	2.67
IFI44L	10964	1.44	<0.05	1.55	<0.05	−0.56	31.00	0.26	2.67
OAS3	4940	1.12	<0.05	0.95	<0.05	−0.43	31.00	0.17	4.16
OAS1	4938	1.05	<0.05	0.89	<0.05	−0.22	36.28	0.18	2.67
IFIT3	3437	1.05	<0.05	0.89	<0.05	−0.66	19.83	0.12	14.21
OAS1	4938	1.04	<0.05	0.86	<0.05	−0.22	36.28	0.18	4.16
OAS2	4939	1.02	<0.05	0.83	<0.05	0.16	49.13	0.15	1.65
IFIH1	64135	1.00	<0.05	0.74	<0.05	0.22	51.55	0.13	11.17
OAS1	4938	1.00	<0.05	0.90	<0.05	−0.20	41.04	0.17	1.65
IFIT1	3434	1.00	<0.05	0.95	<0.05	−0.65	25.32	0.14	14.21
IFIT3	3437	0.90	<0.05	0.83	<0.05	−0.13	45.57	0.18	2.67
OASL	8638	0.90	<0.05	0.69	<0.05	0.23	36.28	0.14	11.17
IFI6	2537	0.89	<0.05	0.71	<0.05	−0.34	31.00	0.05	50.74
OAS2	4939	0.89	<0.05	0.74	<0.05	0.16	49.13	0.11	14.21
DHX58	79132	0.89	<0.05	0.81	<0.05	0.14	53.43	0.20	0.06
IFIT2	3433	0.87	<0.05	0.59	<0.05	−0.34	31.00	0.00	
OAS3	4940	0.83	<0.05	0.79	<0.05	0.11	54.73	0.16	2.67
OASL	8638	0.79	<0.05	0.48	<0.05	0.23	36.28	0.08	11.17
OAS2	4939	0.69	<0.05	0.75	<0.05	−0.39	25.32	0.10	21.71
IFI35	3430	0.62	<0.05	0.64	<0.05	0.13	52.55	0.13	6.18
IFIT5	24138	0.60	<0.05	0.56	<0.05	0.25	45.57	0.12	4.16
IFI16	3428	0.60	<0.05	0.33	<0.05	0.19	15.10	0.06	35.79
IFIT3	3437	0.59	<0.05	0.86	<0.05	−0.66	19.83	0.13	21.71
IFITM3	10410	0.45	<0.05	0.46	<0.05	0.02	NaN	0.16	6.18
IFI27L2	83982	0.45	<0.05	0.39	<0.05	0.55	0.24	0.23	<0.05
IFITM4P	340198	0.44	<0.05	0.39	<0.05	0.19	19.83	0.10	8.35
DHX36	170506	0.44	<0.05	0.30	<0.05	0.40	8.19	0.10	0.95
OAS1	4938	0.41	<0.05	0.57	<0.05	−0.22	36.28	0.11	4.16
OAS2	4939	0.40	<0.05	0.35	<0.05	0.16	49.13	0.05	35.79

Log ratio: Different in expression level between acute and convalescent samples, a value of 0.3 is equal to fold change of 2; FDR: False discovery rate identified by SAM; OFI: Other febrile illness.

#### Down-regulation pathways

**Figure 4:**
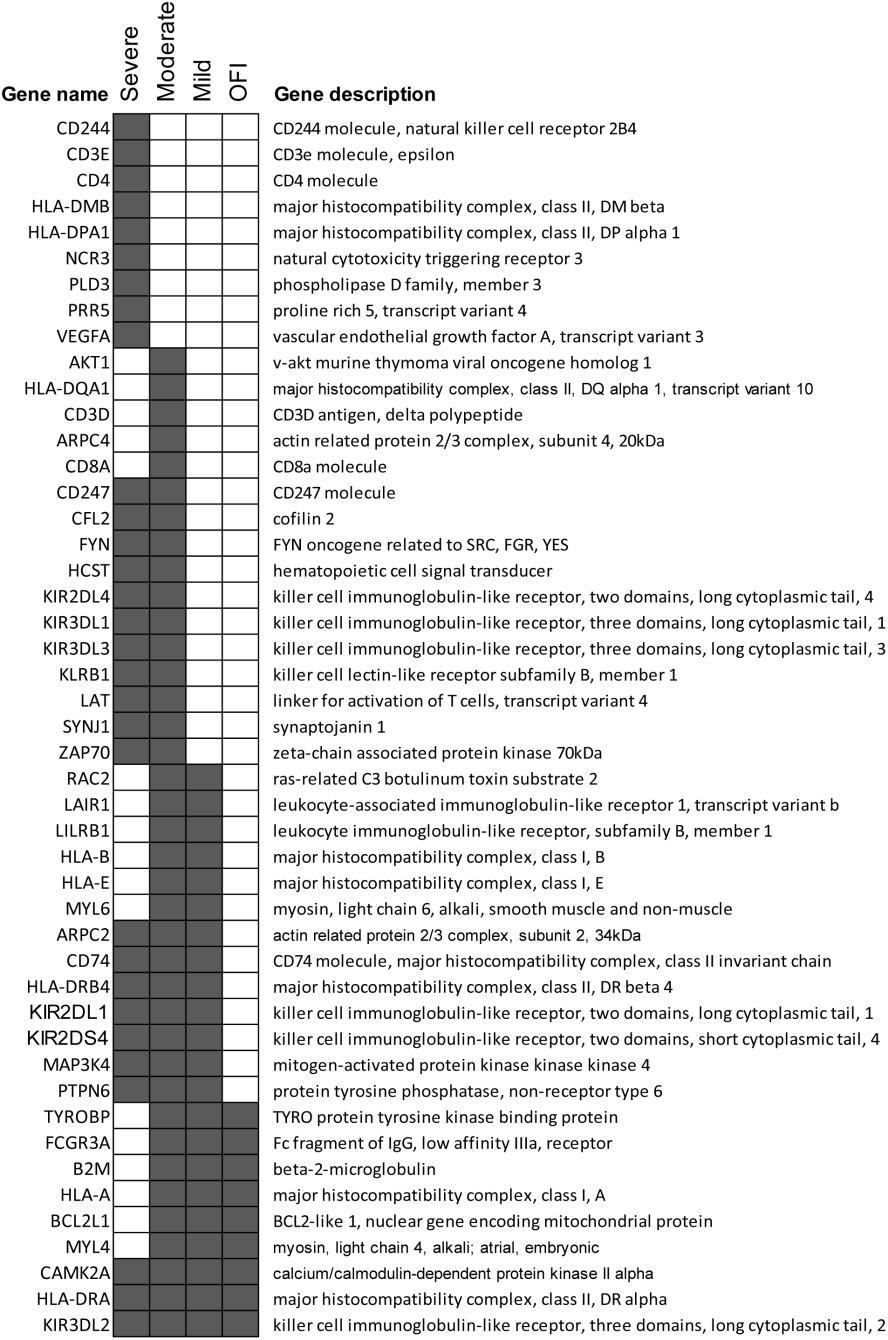
Genes that were involved in the most significantly down-regulated pathways such as Natural Killer Cell Signaling, Crosstalk between Dendritic Cells and Natural Killer Cells, CD28 Signaling in T Helper Cells, PKCθ Signaling in T Lymphocytes. The pathway names were shared between different groups but the activated genes in each pathway were different. Differentially expressed genes (FDR <0.05, fold change >2) were highlighted in grey.

**Figure 5:**
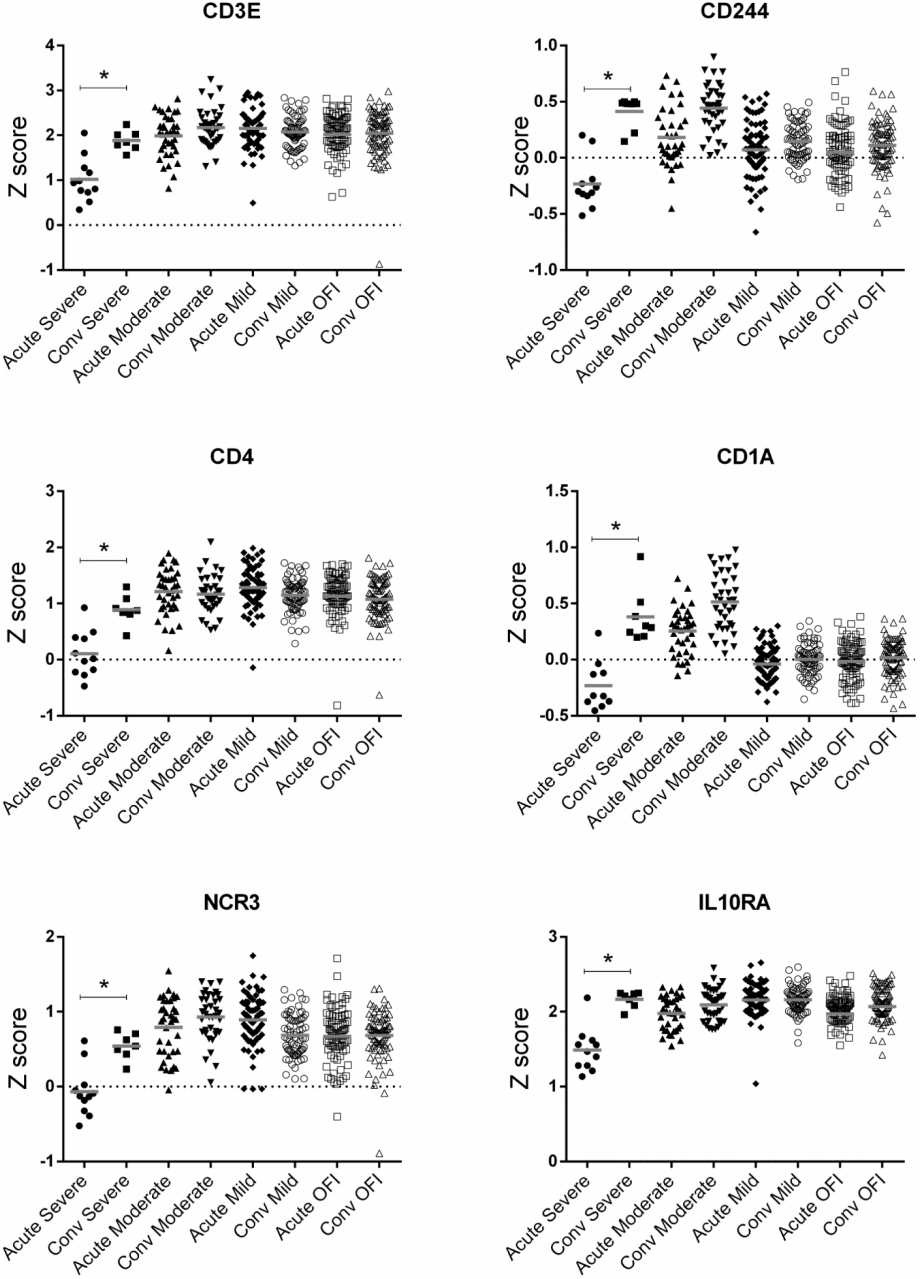
Difference in expression of transcripts in T cell and NK cell signaling pathways. These transcripts were only down-regulated in patients with severe symptoms but not in OFI, mild and moderate disease (*: P≤0.05).

T cell and NK cell related responses were down-regulated in all groups of influenza patients but to a weaker magnitude in those with OFI (**Table 6**). Natural Killer Cell Signaling, Crosstalk between Dendritic Cells and Natural Killer Cells, CD28 Signaling in T Helper Cells, PKCθ Signaling in T Lymphocytes were amongst the most significant pathways. Similar to the up-regulated pathways, although the pathway names were shared between different groups but the genes activated in each pathway were different. NK cell response related genes such as CD247, KIR2DL4, KIR3DL1, KIR3DL3 and KLRB1 were down-regulated only in moderate and severe patients while genes such as KIR2DL1, KIR2DS4 and KIR3DL2 were down-regulated in all three groups of influenza patients (**Figure 4**). CD244, CD3E, CD4, HLA-DMB, HLA-DPA1, NCR3, PLD3, PRR5 and VEGFA were down-regulated only in patients with severe outcomes (**Figure 5**). Beside NK cell and T cells related response, pathways related to the host translational regulation such as eIF4 and p70S6K signaling, EIF2 signaling and mTOR signaling were significantly down-regulated in all influenza infected groups but with a stronger magnitude in patients with mild and moderate disease.

**Table 6:** NK cells and T cells related responses were down-regulated in patients with severe, moderate and mild outcomes but not in patients with OFI.

Ingenuity Canonical Pathways	Severe			Moderate			Mild			OFI		
	P	Ratio	# genes	P	Ratio	# genes	P	Ratio	# genes	P	Ratio	# genes
Natural Killer Cell Signaling	9.0	0.14	16	5.3	0.17	20	2.6	0.09	11	0.6	0.03	3
Crosstalk between Dendritic Cells and Natural Killer Cells	3.4	0.08	9	5.3	0.17	18	4.4	0.12	13	2.3	0.06	6
CD28 Signaling in T Helper Cells	3.4	0.07	10	0.7	0.08	11	0.6	0.04	6	0.2	0.01	1
PKCθ Signaling in T Lymphocytes	3.4	0.07	10	0.5	0.07	10	0.2	0.03	4	0.3	0.01	2
EIF2 Signaling	3.1	0.06	12	43.6	0.35	70	35.2	0.25	51	1.2	0.03	6
iCOS-iCOSL Signaling in T Helper Cells	2.2	0.06	8	0.3	0.06	8	0.1	0.02	3	0.3	0.02	2
Antigen Presentation Pathway	2.2	0.12	5	2.1	0.19	8	3.0	0.17	7	1.2	0.07	3
OX40 Signaling Pathway	2.2	0.07	7	1.0	0.10	10	1.3	0.07	7	1.1	0.04	4
Calcium-induced T Lymphocyte Apoptosis	2.2	0.08	6	0.2	0.07	5	0.1	0.03	2	0.2	0.01	1
mTOR Signaling	2.0	0.05	10	14.4	0.19	41	12.7	0.14	30	0.2	0.01	2
Role of NFAT in Regulation of the Immune Response	1.7	0.05	9	0.8	0.08	15	1.1	0.05	10	0.2	0.01	2
CTLA4 Signaling in Cytotoxic T Lymphocytes	1.5	0.06	6	4.1	0.17	16	1.3	0.07	7	0.4	0.02	2
Cdc42 Signaling	1.4	0.04	8	0.8	0.08	14	1.6	0.06	11	0.7	0.02	4
T Cell Receptor Signaling	1.4	0.06	6	0.1	0.06	6	NS	0.00	0	NS	0.00	0

## Discussion

By investigating the transcriptional profiles of patients with a wide range of disease severity from non-influenza fever of unknown aetiology to mild, moderate and severe influenza, we found that patients with confirmed influenza infections had similar and stronger host responses in comparison with patients with fever of unknown aetiology. The interferon response was shown to be strongly up-regulated in influenza virus A infection [11], however, the exact mechanism of interferon responses in different clinical severities of influenza has not been described. Here, we found that the interferon signaling pathway and the protein ubiquitination pathway were attenuated in patients with fever of unknown aetiology and patients with severe influenza manifestations suggesting the protective roles of these pathways in the disease mechanism. Furthermore, our data suggests that interferon signaling pathway and other interferon-induced transmembrane proteins (IFITMs) play important protective roles in the disease mechanism. Amongst the IFITMs, IFITM3 has been shown to block influenza virus entry to the cells [12] and limit the severity of influenza virus infection in mice [13]. Our data suggests that not only IFITM3, but other IFITMs and ISGs genes could similarly play important roles in the disease outcomes.

Apart from the interferon pathway, the ubiquitiniation signaling pathway was also attenuated in patients with severe outcome. Protein ubiquitination is a post-translational process involving the addition of ubiquitin to a protein substrate, and it plays important roles in protein-protein interactions. For example the NS1 protein from influenza A virus could inhibit the IFN response by inhibiting the ubiquitination of the viral RNA sensor RIG-I [14]. RIG-I is the key host sensor for viral RNA inside the cytoplasm that accounts for the induction of the host IFN response [15]. Ubiquitination of the CARD domain in RIG-I is essential for the activation of IRF3 and NFkB which in turn induce the IFN production [16] [17]. As mentioned above, ubiquitination of RIG-I is the key process by which the host could recognize the influenza virus RNA to induce interferon response. The attenuation of the protein ubiquitination pathway and RIG-I could lead to the lack of interferon response in the severe patients. It was also shown that the host protein ubiquitination system is required for influenza virus replication [17]. This may explain why the percentage of patients who had virus detected at sample collection was lower in the severe group (55%) in comparison with those in the moderate group (86%).

As mentioned, although the same pathways were up-regulated in the patients with different severity outcomes, certain genes such as TLR10, NFKBIA, IL1R2, SOCS3, IL4R, IL1R1, PROK1, ECE1, IFNAR1, MMP9, PPP1R10 and PPP2R2A were up-regulated only in the severe patients. It was shown in mouse model that MMP9, which is capable of digesting the extracellular matrix, was produced mainly by neutrophils and was required for neutrophils migration to the respiratory tract in response against viral replication [18]. The up-regulation of MMP9 in the severe patients suggests the contribution of this gene in the disease outcome. The up-regulation of the Suppressor of cytokine signaling 3 (SOCS3) gene was IFNAR1-dependent and it has negative regulatory functions to the innate immune response which could inhibit type I interferon signaling [19]. The lack of interferon signaling in the severe patients could be explained by the up-regulation of SOCS3 in these patients. TLR10 has recently been shown to play a role in innate immune response in influenza infection and that its expression is stronger during infection by the highly pathogenic influenza H5N1 virus [20].

Amongst the down-regulated pathways, the T cell signaling response and NK cell responses were predominant in all influenza patients. More interestingly, these pathways in patients with severe disease were more predominant in comparison with patients with mild and moderate disease. Previous studies have shown that type I interferon response could be exploited by opportunistic pathogens in influenza infection which in turn could increase the host susceptibility to secondary bacterial infections such as *Streptococcus pneumoniae* by negatively regulating the T cell response [21]. We did not detect any secondary bacterial infections in the moderate and severe patients by using both conventional blood culture or by 16S rRNA sequencing (data not shown). This could be due to the fact that all of the patients were treated with antibiotics before they were recruited to the study.

Many genes involved in T cell and NK cell responses were down-regulated in influenza infected patients regardless of their clinical outcome, however, we found that some keys transcripts in the T cell and NK cell signaling response were only down-regulated in patients with severe influenza (CD244, CD3E, CD4, CD1A, NCR3 and IL10RA) suggesting the important roles of these transcripts in severe influenza. Previous studies have shown that severe influenza A disease was associated with a transient NK cell and CD8 T cell response [22], [23]. Amongst the down-regulated genes, NCR3 has been reported to show a direct interaction with influenza viruses whereby the virus down-regulates the cytotoxicity of NK cells mediated by this gene [24], [25]. In addition, single polymorphisms in *NCR3* have been shown to be strongly associated with decreased lung function in a recently conducted large scale (N>48,000) genome-wide association study of forced expiratory volume as a surrogate for lung function [26].

Despite having relatively large sample sizes for the moderate, mild and OFI groups, the one limitation of our study is the small sample size of severe influenza patients that we managed to recruit in the duration of the study. Nevertheless, we have generated a large exploratory transcriptomic dataset in influenza disease with a wide spectrum of severity. Although the validation of these findings is beyond the scale of the current study, our dataset can serve as a valuable data mine for researchers in the field to select their own gene candidates for hypothesis testing and validation. Taken together, by investigating the global host transcriptional profile in influenza virus A infected patients with different clinical outcomes, we have provided insights into the global gene expression in influenza virus A infection. In particular, we have revealed the attenuation of interferon and protein ubiquitination pathways and the down-regulation of T and NK cell related responses in patients with severe influenza virus A infections. Lastly, we propose the following candidate genes for further studies: MMP9, SOCS3, IFITMs, TLR10, RIG-I, CD244 and NCR3.
